# The complete chloroplast genome of *Holboellia angustifolia* (Ranales: Lardizabalaceae), a traditional herbal species

**DOI:** 10.1080/23802359.2019.1674214

**Published:** 2019-10-04

**Authors:** Xiaozhu Wu, Tongjian Li, Feng Wen

**Affiliations:** College of Pharmacy and Life Sciences, Jiujiang University, Jiujiang, Jiangxi, People’s Republic of China

**Keywords:** Complete chloroplast genome, *Holboellia angustifolia*, Illumina sequencing, phylogenotic analysis

## Abstract

The complete chloroplast genome of *Holboellia angustifolia* was 157,797 bp in length, displayed a typical quadripartite structure, composed of a LSC region of 86,543 bp and a SSC region of 18,972 bp, separated by a pair of IRs of 26,141 bp each. The chloroplast genome contains 130 genes, consisting of 85 protein-coding genes, 37 tRNA genes, and 8 rRNA genes. Fifteen genes have one intron, and 3 genes contain two introns. The overall A/T content in the chloroplast genome of *H. angustifolia* was 61.31%. Phylogenetic analysis showed that *H. angustifolia* was closely related to *Holboellia latifolia*.

*Holboellia angustifolia* Wall., belonging to the family Lardizabalaceae, is an evergreen wood vine widely distributed in the hillside and valley forest in the southern region of China, including Jiangxi, Yunnan, Guizhou, Guangdong, and Guangxi provinces (Flora of China Editorial Committee 2001). Its stem, root, and fruit have been utilized as traditional herbal medicine for the treatment of cough, lumbago, nephritis, and abdominal hernia (Fu et al. 2001). Previous phytochemical investigation on *H. angustifolia* revealed that five triterpenoid saponins, fargosides A–E, were potential medicinal compounds from the roots of *H. angustifolia* (Fu et al. 2001). In addition, the seeds of *H. angustifolia* contain nearly 30% oil with high linoleic acid content, which could be considered as a potential oil crop (Lei et al. 2009). Here, the complete chloroplast genome of *H. angustifolia* was determined and characterized, based on Illumina sequencing data.

In this study, fresh leaves of *H. angustifolia* was sampled from Lengquan town in Mengzi city (113.23E,23.13N), Yunnan Province, China. The voucher specimen deposited in Jiujiang University (accession number JJU130829). Total genomic DNA was isolated from ∼5 g of fresh leaves for harvesting cpDNA using an improved extraction method (McPherson et al. 2013). Short-insert libraries (insert size 430 bp) constructed according to the manufacturer’s instructions (Illumina), and then sequenced on the Illumina Hiseq 4000 platform at BIOZERON Co., Ltd. (Shanghai, China) (Borgstrom et al. 2011). Raw reads were filtered and then assembled into contigs via SOAPdenovo 2.04 (Luo et al. 2012), using the *Holboellia latifolia* chloroplast genome as a reference (GenBank accession NC_039622). The finished chloroplast genome was annotated using an online DOGMA tool (Wyman et al. 2004), using default parameters to predict protein-coding genes, transfer RNA (tRNA) genes, and ribosome RNA (rRNA) genes, coupled with manual check and adjustment.

The whole chloroplast genome size of *H. angustifolia* was 157,797 bp in length, displayed a typical quadripartite structure, which consisted of a pair of inverted repeats (IRs, including IRa and IRb) of 26,141 bp, separated by a large single-copy (LSC) region, and a small single-copy (SSC) region of 86,543 and 18,972 bp, respectively. The chloroplast genome contains 130 genes, consisting of 85 protein-coding genes (79 PCG species), 37 tRNA genes (30 tRNA species), and 8 rRNA genes(4 rRNA species). Most chloroplast genes occurred as a single copy, however, 17 genes were duplicated in the IR region, including six PCGs (*ndhB*, *rpl2*, *rpl23*, *rps7*, *rps12*, and *ycf2*), seven tRNA genes (*trnA-UGC*, *trnI-CAU*, *trnI-GAU*, *trnL-CAA*, *trnN-GUU*, *trnR-ACG*, and *trnV-GAC*), and all four rRNA genes (*rrn23*, *rrn16*, *rrn5*, and *rrn4.5*). Twelve PCG genes contained introns, nine of them (*atpF*, *ndhA*, *ndhB*, *petB*, *petD*, *rpl2*, *rpl16*, *rpoC1*, and *rps16*) harboured a single intron and three genes (*clpP*, *rps12* and *ycf3*) exhibited two introns. Six tRNA genes (*trnA-UGC*, *trnG-GCC*, *trnI-GAU*, *trnK-UUU*, *trnL-UAA*, and *trnV-UAC*) contained one intron. The nucleotides composed of 30.31% A, 19.68% C, 19.01% G, and 31.01% T. The overall A/T content was 61.32%, while the corresponding values of LSC, SSC, and IR regions were 62.88, 66.27, and 56.91%, respectively.

To investigate the phylogenetic status of *H. angustifolia*, a maximum-likelihood (ML) phylogenetic tree including *H. angustifolia*, five Lardizabalaceae species, and the outgroup *Euptelea pleiosperma* was constructed by complete chloroplast genomes, by using the software MEGA-X with 1000 bootstrap replicates (Kumar et al. 2018). As shown in the phylogenetic tree (Figure 1), *H. angustifolia* was the most closely related to *Holboellia latifolia*. In this study, we reported and characterized the complete chloroplast genome sequence of *H. angustifolia* as a resource, which would be useful for future phylogenetic and population genomic studies for family Lardizabalaceae.

**Figure 1.**
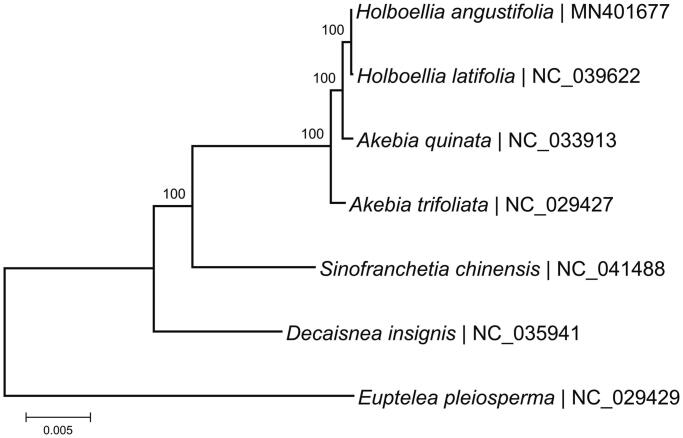
Maximum-likelihood (ML) tree based on the complete chloroplast genome sequences of 7 species. The numbers on the branches are bootstrap values.
